# Whisker Contact Detection of Rodents Based on Slow and Fast Mechanical Inputs

**DOI:** 10.3389/fnbeh.2016.00251

**Published:** 2017-01-10

**Authors:** Laure N. Claverie, Yves Boubenec, Georges Debrégeas, Alexis M. Prevost, Elie Wandersman

**Affiliations:** ^1^Sorbonne Universités, UPMC Univ Paris 06, UMR 8237, Laboratoire Jean PerrinParis, France; ^2^Laboratoire des Systèmes Perceptifs, Département d'études Cognitives, ENS, PSL Research University, Centre National de la Recherche ScientifiqueParis, France

**Keywords:** rodents tactile perception, whisker contact detection, whisking, whiskers slow deformations, whiskers fast vibrations

## Abstract

Rodents use their whiskers to locate nearby objects with an extreme precision. To perform such tasks, they need to detect whisker/object contacts with a high temporal accuracy. This contact detection is conveyed by classes of mechanoreceptors whose neural activity is sensitive to either slow or fast time varying mechanical stresses acting at the base of the whiskers. We developed a biomimetic approach to separate and characterize slow quasi-static and fast vibrational stress signals acting on a whisker base in realistic exploratory phases, using experiments on both real and artificial whiskers. Both slow and fast mechanical inputs are successfully captured using a mechanical model of the whisker. We present and discuss consequences of the whisking process in purely mechanical terms and hypothesize that free whisking in air sets a mechanical threshold for contact detection. The time resolution and robustness of the contact detection strategies based on either slow or fast stress signals are determined. Contact detection based on the vibrational signal is faster and more robust to exploratory conditions than the slow quasi-static component, although both slow/fast components allow localizing the object.

## 1. Introduction

Rodents use their facial whiskers (vibrissa) to locate objects (Krupa et al., 2001; Knutsen, 2006; Mehta et al., 2007; O'Connor et al., 2010), apprehend their shape (Brecht et al., 1997; Polley et al., 2005; Anjum et al., 2006) and texture (Carvell and Simons, 1990; Lottem and Azouz, 2008; Boubenec et al., 2014) with an extreme precision. The vibrissal system is a model system in neuroscience to study the processing of sensory inputs, elicited by the whisker/object contacts. In a localization task (Krupa et al., 2001; Pammer et al., 2013; Voigts et al., 2015), contacts occur as a result of both rodent's body or head movements, combined with periodic whisker retraction/protraction cycles elicited by an active motor pattern called *whisking*. The whisker being sensorless, contact induced deflections are detected by mechanoreceptors embedded in the follicle, at the whisker base. Different types of mechanoreceptors have been identified (Szwed et al., 2003; Szwed, 2005), sensitive to either *slow* (Slowly Adapting, SA) or *fast* (Fast Adapting, FA) time varying stresses. SA mechanoreceptors respond to a steady applied stress while FA mechanoreceptors are triggered by rapid events. Such rapid events, produced for instance by contact-detachment (Szwed et al., 2003; Lottem and Azouz, 2009) and frictional stick-slip (Ritt et al., 2008) trigger enhanced neural activity along the trigeminal pathway.

Within this mechanosensory framework, two neural codings, possibly complementary, have been proposed (Panzeri et al., 2001; Petersen et al., 2001; Johansson and Flanagan, 2009). The rate-coding scenario proposes that the stimulus intensity is encoded in the firing rate of the afferent neurons, likely involving SA mechanoreceptors. The second coding relies on the timing of single spikes at short timescales (Panzeri et al., 2001; Petersen et al., 2001), presumably involving FA mechanoreceptors. The former requires a measure of the interspike frequency, whose upper bound is set by the kinetics of the internal mechanoreceptors membrane depolarization (Louhivuori et al., 2010). Moreover, a sufficient number of spikes are required to properly estimate the firing rate. This limits the efficiency of such coding to timescales of about 10 ms or more (Shoykhet et al., 2000) and thus its involvement in slow processes. On the other hand, the spike timing scenario requires coincidence measurements of multiple afferent neurons activity. It has been shown that a whisker/object contact triggers activity in the trigeminal ganglion after 1 ms (Bale et al., 2015), with a jitter of about tens of microseconds, less than the interspike delay. Such temporal precision favors a spike timing scenario for first contacts detection.

From a mechanical point of view, it thus appears important to separate and characterize the *slow/fast* mechanical signature at the whisker base elicited during a typical exploration sequence. Such measurements are however difficult in real conditions, since head/body movements and whisking produce complex stress signals. It calls for simpler experimental approaches. For the detection and localization of an object, attempts have been made to model and measure the quasi-static deformations of the vibrissae (Hartmann et al., 2003; Solomon and Hartmann, 2008). Hartmann et al. derived the quasi-static base torque signal when the whisker is indented by an object and show that it can be used to localize an object (Birdwell et al., 2007; Solomon and Hartmann, 2008, 2010). However, an experimental validation of this model on real whiskers is lacking. Besides the quasi-static deformations, the whisker has specific vibrational properties (Neimark et al., 2003). We have shown previously (Boubenec et al., 2012, 2014) that the contact triggers a deflection wave that propagates down to the base, inducing shortly after the shock, characteristic modulations of the base torque. This fast signal could be used as a mechanical input for spike timing coding strategies.

In this paper, we report experimental investigations and theoretical modeling of both the quasi-static and the dynamical stresses acting on the whisker base of a whisker contacting a sharp object. We use a biomimetic approach and consider an artificial whisker attached to a torque sensor and imaged with a fast camera. We perform experiments using simple whisker/object displacements (whisker steady rotation, object uniform translation, sinusoidal whisking) to characterize the quasi-static and dynamical mechanical stresses. We show that both components of the base torque allow a radial localization of the object and provide a first full experimental validation of the quasi-static model of Hartmann et al. (2003), Solomon and Hartmann (2008) with artificial/real whiskers, along with a first experimental validation of the dynamical model of Boubenec et al. (2012). Finally, we mimic a simple exploration task involving whisking and discuss the efficiency of quasi-static vs. dynamical contact detection strategies.

## 2. Materials and methods

### 2.1. Experimental setup

**Figure 1:**
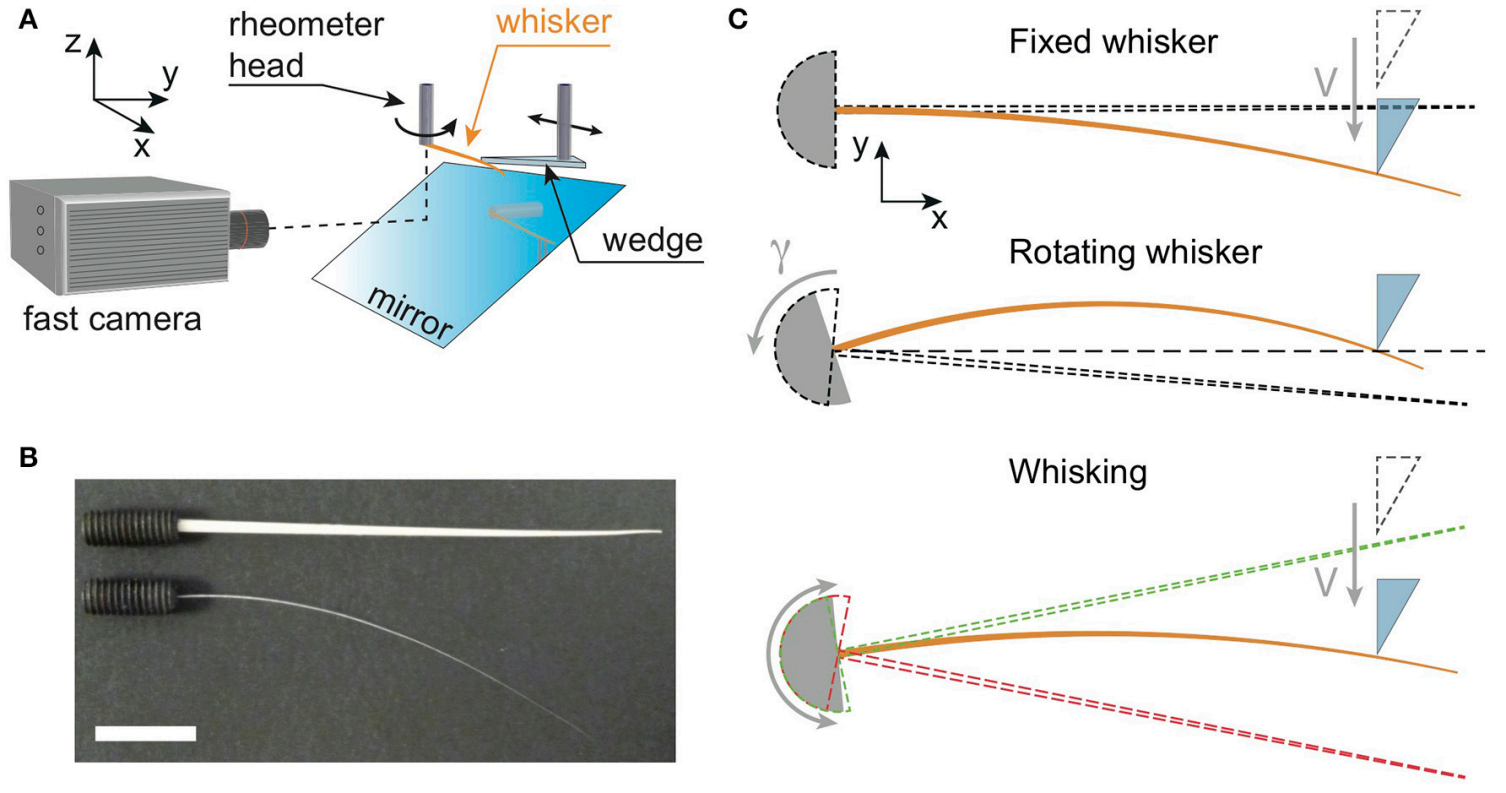
**(A)** Sketch of the experimental setup. Whiskers (orange color), either real or artificial, are attached at their base to the cylindrical shaft of a rheometer head which measures the component of the base torque normal to the (*x, y*) rotation plane. They can be maintained at a fixed base angle, and hit by a Plexiglas wedge mounted on a rotary motor, or alternatively rotated at a constant angular velocity while the wedge remains fixed (as shown on this sketch). The rotary motor is itself fixed to a linear rail, so that the contact point location can be moved along the whisker. In addition, a high speed camera records the whisker deflections in the rotation plane *via* a mirror. **(B)** Picture showing both artificial (top) and real (bottom) whiskers glued at their base to drilled studs. The white bar is 1 cm long. **(C)** Three types of experiments. From top to bottom: *Fixed whisker experiment*—the base angle is fixed while the wedge indents at constant velocity *V* the whisker whose initial position is shown with the dashed line cone; *Rotating whisker experiment—* the base angle of the whisker (initial position shown with the dashed line cone) is rotated at a constant angular velocity γ against a fixed wedge; *Whisking experiment—*the base angle oscillates sinusoidally between two extreme positions (red and green dashed line cones), while the wedge indents the whisker at constant *V*.

We have reproduced typical radial localization tasks performed by rodents using a minimal biomimetic setup (Figure 1A) which involves the use of an MCR 302 Anton Paar rheometer (a highly sensitive torque sensor mounted on an air bearing rotating motor). A single whisker, either real or artificial is glued at its base with cyanoacrylate to a stud (Figure 1B) attached to the rotating axis of the rheometer head. The rheometer head, and its embedded torque sensor, is used to mimic a single mechanoreceptor in the base follicle. It allows measuring the torque exerted at the base of the whisker, along the direction normal to the rotation plane of the whisker. Since the base angle of the whisker and its angular velocity can be set by the head of the rheometer, various exploration modes can be reproduced ranging from a fixed whisker whose base angle remains constant or a rotating whisker with a constant angular velocity to an oscillating whisker. To simulate a shock with an object, whiskers can be indented locally at a distance *d* from their base, with a Plexiglas wedge (sometimes also referred to as the “indenter”) to produce a quasi point-like contact.

#### 2.1.1. Artificial and real whiskers

We designed an artificial whisker made from PolyUrethane (PU) using a molding technique. In a first step, a brass cone is obtained by pulling at constant velocity (~ 1 mm.s^−1^) a brass cylindrical rod (K & S Precision Metals) of diameter 1.5 mm, from a 1:1 nitric acid-water solution (Lorenceau and Quéré, 2004). The pulling velocity can be tuned to adjust the conicity of the whisker. In a second step, the brass cone is molded in a PolyVinylSiloxane resin (Elite Double 8, Zhermack SpA, Italy) which crosslinks at room temperature in about tens of minutes. The brass cone is then carefully removed, yielding a void that is later filled with a liquid PU resin. After polymerization, the resulting PU whisker has the same conical shape as the brass cone. Real whiskers were cut from a 4-month old Sprague-Dawley male rat. For the present study, only C1 whiskers were used. Experiments were conducted in conformity with French (JO 2001-464) and European legislation (86/609/CEE) on animal experimentation.

**Figure 2:**
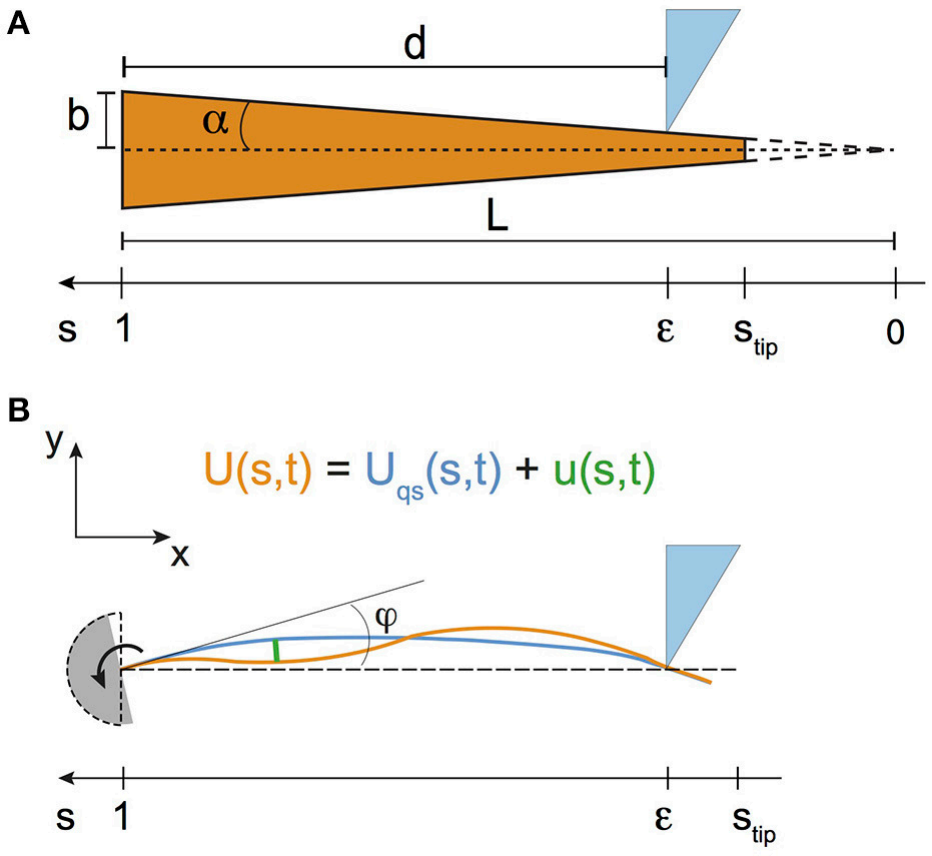
**(A)** Sketch of the whisker, modeled as a truncated cone of base radius *b* and conicity α. **(B)** Modeling the whiskers displacements in a shock experiment, for the case of a rotating whisker and a fixed indenter. The base angle φ is taken from the undeformed vertical position of the whisker (shown with the dashed lines).

Geometrical and mechanical properties of both types of whiskers are shown in Table 1. Both base radius *b* and conicity α (see their definition in Figure 2A) were obtained by image analysis of high resolution pictures of the whiskers taken with a high magnification binocular microscope. The Young's modulus *E* of the artificial whisker was obtained by measuring, in the elastic regime, the extensional deformation of cylindrical PU rods using an Instron 5565 tensile tester. The mass density of the artificial whisker ρ was determined by weighing cylindrical PU rods of different lengths. For the real whisker, typical values of both *E* and ρ were taken from references Quist et al. (2011) and Hartmann et al. (2003), respectively.

**Table 1:** **Whiskers geometrical and mechanical properties**.

	**Artificial**	**Real**
b (μm)	600 ± 14	88 ± 1
α (mrad)	8.1 ± 0.4	1.8 ± 0.1
*L* (cm)	7.4 ± 0.4	4.9 ± 0.3
*E* (GPa)	1.19 ± 0.03	3.3 ± 1.5
ρ (10^3^ kg/m^3^)	1.14 ± 0.04	1.1 ± 0.3

*For the real whisker, the values of E and ρ are taken respectively from Quist et al. (2011) and Hartmann et al. (2003)*.

#### 2.1.2. Base torque and whisker deflections measurements

In typical low shock velocity regimes, base torque signals are directly measured with the rheometer head with a ~ 1 μNm resolution. In some cases however, base torque signals could not be measured with it, being below of either the rheometer's time or amplitude resolution. This is the case when the object hits the whisker at high shock velocities, where base torque signals vary too fast to be measured. It is also the case when the object hits the fixed real whisker far from its base (typically when *d* > 2 cm), resulting in amplitudes of the base torque signals often too low to be detected. For both situations, base torques are thus obtained using image analysis of the whiskers profiles, as detailed further down (see Section entitled “Whisker detection using image analysis”). Whiskers profiles are recorded using a high speed camera (Fastcam APX-RS, Photron, full frame 1024 × 1024 pixels^2^, 8 bits) operating at frame rates *f*_*cam*_ in the range [500–25,000] frames per second (fps), with an exposure time set by the inverse of the frame rate. The whisker is illuminated in reflection using a white light halogen lamp (Leica CLS 150X) combined with an optical fiber. As a result, whiskers appear bright on a darker background. Images of the whisker on the CMOS sensor of the camera are obtained with the combination of a mirror, positioned at an angle of 45° with respect to the rotation axis (Figure 1A), and a macro-objective (105 mm, f-2.8, DG Macro, Sigma), yielding a pixel size of about 60 μm.

#### 2.1.3. Shock experiments

Three different types of experiments have been performed and are reported in this work (Figure 1C).

- *Fixed whisker experiments*: The base angle of the whisker is maintained at a fixed value, while the indenter collides with the whisker at a distance *d* from the base with a prescribed shock velocity *V*. For these experiments, the indenter is mounted orthogonally to the direction of the shaft of a rotary stepper motor (Model 23HSX-206, Mclennan Servo Supplies Ltd., UK) allowing to explore a large range of shock velocities. The stepper motor itself is mounted on a linear rail which is used to set manually the distance *d* (Figure 1C top panel).
- *Rotating whisker experiments*: The whisker rotates around the rheometer axis at a constant angular velocity $\gamma =\frac{d\varphi }{dt}$ and hits the fixed indenter. The point of contact is similarly set manually using the linear rail (Figure 1C, middle panel).
- *Whisking experiments* : The whisker oscillates around the rheometer axis at a frequency *f*_*w*_ = 15 Hz and amplitude φ_0_ = 10°, to mimic the whisking motion of rodents. To reproduce in the most basic way the approach of rodents toward an obstacle, the indenter is mounted on a linear motorized translation stage (DDS220/M, Thorlabs Inc., USA). The initial position of the indenter is out of reach of the whisker, so that the first phase of the experiment consists in a free whisking in air. The indenter is then translated at a constant velocity toward its final position where it is stopped. The last phase of the experiment thus consists of multiple successive contacts with the fixed indenter (Figure 1C, bottom panel).

In all three experiments, all elements (rheometer head, fast camera, stepper motor and motorized translation stage) are synchronized and interfaced using a LabVIEW (National Instruments Corp., USA) custom made routine.

### 2.2. Whisker detection using image analysis

Whenever direct base torque measurements with the rheometer head sensor were not possible (as already explained earlier in the text), base torque signals were obtained by image analysis of the spatial profiles of the whiskers. As detailed further down (see Section ‘Modeling the whisker dynamics’) the base torque *M* is proportional to the base curvature *C* of the whisker. The base curvature is determined by measuring the whisker displacements, using image analysis as follows.

Since the whiskers displacements are small, they can be in good approximation assimilated to the displacement along the *y* axis (see Figure 1C). Positions of the whisker are deduced with a sub-pixel resolution from the intensity barycenter along each column of the images. Profiles are then interpolated on a curvilinear coordinate system. We thus obtain the whisker coordinates *y*(*s, t*) (with *s* the curvilinear coordinate) from which the transverse displacement *U*(*s, t*) is computed. Depending on the type of experiment, computation of *U*(*s, t*) is slightly different.

- *Fixed whisker experiments*: The displacement *U*(*s, t*) is computed at every instant *t* as the difference between the actual whisker spatial profile and an unconstrained reference one. Profiles are further smoothed on a 10 pixel wide (~600 μm) sliding window and fitted by a third order degree polynomial with the constraint that the spatial derivative of the displacement must equal zero at the base (to take into account the rigid anchoring at the base).
- *Whisking experiments*: The method just described could not resolve minute inertia-induced displacements, and *U*(*s, t*) was computed by considering the time evolution of *U*(*s, t*) for fixed discrete positions *s* along the whisker's profile *y*(*s*). For each whisking experiment, which consisted in oscillating the base of the whisker with a maximum amplitude φ_0_ and an angular frequency of 15 Hz, a twin reference experiment was performed. It consisted in rotating the whisker at a very low angular velocity (with a whisking frequency *f*_*w*_ of 0.5 Hz) and same φ_0_. In this case, inertia effects vanish and the motion of the whisker in air is purely solid-like. In free air (no contact), the profile's position *y*_*slow*_(*s*) thus evolves sinusoidally with time, and fitting this curve with a sine wave yields its amplitude *a*_*slow*_(*s*). Similarly, for the actual whisking experiment at 15 Hz, *y*(*s*) curves in free air at all times are fitted with a sine wave yielding an amplitude *a*_*fast*_(*s*), a pulsation ω_*fast*_(*s*) and a phase ϕ_*fast*_(*s*). The displacement *U*(*s, t*) at each time *t* in free air and during contacts is then taken as *U*(*s, t*) = *y*(*s*) − *a*_*slow*_(*s*)sin(ω_*fast*_(*s*)*t*+ϕ_*fast*_(*s*)). *U*(*s, t*) is further fitted by a third order degree polynomial imposing that the spatial derivative of the displacement is equal to zero at the base.

Once the whiskers displacement *U*(*s, t*) have been obtained, the curvature *C*(*s, t*) = ∂^2^*U*(*s, t*)/∂*s*^2^ is computed, from which the base torque *M* can be deduced (see Section ‘Results’).

### 2.3. Modeling the whisker dynamics

Whiskers are treated as elastic truncated cones with no intrinsic curvature (Figure 2A). Their untruncated lengths *L* are deduced from the ratio of their base radius *b* to their conicity α, i.e., *L* = *b*/α. In the following, all lengths are normalized by *L*. The curvilinear coordinate *s* thus takes the values 0 at the (virtual) tip and 1 at the base. In addition, the curvilinear coordinate ϵ at contact between the whisker and the object is related to the radial distance *d* by the relationship ϵ = 1 − *d*/*L*.

The time evolution of the whisker profile is characterized by its transverse displacement with respect to its undeformed profile (as defined in Figure 2B). This quantity, noted *U*(*s, t*) obeys the Euler-Bernoulli's equation, which reads, for a cone (Boubenec et al., 2012)

(1) $$\frac{\partial^{2}}{\partial s^{2}}\left(s^{4}\frac{\partial^{2}U(s,t)}{\partial s^{2}}\right)+k^{2}s^{2}\frac{\partial^{2}U(s,t)}{\partial t^{2}}=0$$

where $k=2\sqrt{\rho /E}L/\alpha$ is a timescale that characterizes the mechanical resonance of the isolated whisker. Solving for Equation (1) with the appropriate boundary conditions yields the dynamic profile *U*(*s, t*). Since the whisker deformation is planar in the (*x, y*) plane, the relevant mechanical quantity is the *z* component of the base torque *M*(*s* = 1, *t*). The latter is computed as follows

(2) $$M(s=1,t)=E\;I(s=1)\;C(s=1,t)=\frac{E\pi b^{4}}{4}\;\frac{1}{L^{2}}{\frac{\partial^{2}U(s,t)}{\partial s^{2}}|}_{s=1}$$

In Equation (2), *I* is the area moment of inertia whose value at the base *I*(*s* = 1) = π*b*^4^/4, and $C=\frac{1}{L^{2}}\frac{\partial^{2}U}{\partial s^{2}}$ is the curvature in the limit of small deflections. Following Boubenec et al. (2012), *U*(*s, t*) is written as the sum of a quasi-static term *U*_*qs*_(*s, t*) and a dynamic one *u*(*s, t*) (Figure 2B)

(3) $$U(s,t)=U_{qs}(s,t)+u(s,t)$$

Similarly, the base torque *M*(*s* = 1, *t*) is the sum of a quasi-static part and a dynamic one. From here on, the total base torque *M*(*s* = 1, *t*) will be referred to as *M*(*t*), while its quasi-static component will be denoted *M*_*qs*_(*t*).

Within this theoretical framework, we now derive predictions for both quasi-static and dynamic whiskers deflections and related base torques, in two situations that are relevant for the present work, namely for *quasi point-like shock experiments* and for the *free whisking in air*.

#### 2.3.1. Shock experiments

Both shock configurations are considered here, namely the fixed whisker experiment (an object with a velocity V encounters the whisker at *s* = ϵ) and the rotating whisker experiment on a fixed object (the whisker rotates at a constant angular velocity $\gamma =\frac{d\varphi }{dt}$ where φ is the protraction angle, and contacts the object at *s* = ϵ). In both cases, the instant of the shock is arbitrarily taken at *t* = 0.

- Quasi-static deflections
  After the shock (*t* > 0) and in the limit of small deflections (Boubenec et al., 2012), *U*_*qs*_ is obtained by solving for each set of boundary conditions corresponding to both type of experiments (see Table 2) the following equation
  (4) $$\frac{\partial^{2}}{\partial s^{2}}\left(s^{4}\frac{\partial^{2}U_{qs}(s,t)}{\partial s^{2}}\right)=0$$
  Equation (4) can be solved analytically, yielding *U*_*qs*_(*s, t*) and *M*_*qs*_(*t*), given in Table 2. Note that the quasi-static term *U*_*qs*_(*s, t*) characterizes equilibrium deflections in the low velocity regime, and that its magnitude is proportional to the indentation amplitude, *Vt* for the fixed whisker experiment and φ = γ*t* for the rotating whisker experiment.
- Shock induced dynamic deflections
  Following (Boubenec et al., 2012), the dynamic part of the deflections *u*(*s, t*) can be decomposed onto the spatial eigenmodes *V*_*i*_ as $u(s,t)=\sum_{i}\;q_{i}(t)\;V_{i}(s)$, with amplitudes *q*_*i*_. As shown in Boubenec et al. (2012), the eigenmodes *V*_*i*_ are solution of the following equation
  (5) $${\left(s^{4}V_{i}{}^{\prime \prime }\right)}^{\prime \prime }-k^{2}\omega_{i}^{2}s^{2}V_{i}=0$$
  with the appropriate boundary conditions. In Equation (5), the ω_*i*_ are the eigenfrequencies of the spatial eigenmodes *V*_*i*_ and the symbol ″ is meant for ∂^2^/∂*s*^2^ (similarly further down, ′ and ^‴^ stand for the first and third spatial derivatives with respect to *s*). In the case of a quasi point-like shock, the whisker is considered to be fixed at its base *s* = 1 and pinned at the contact point with curvilinear coordinate *s* = ϵ. Boundary conditions thus read *V*_*i*_(1) = 0, $V_{i}^{\prime }\left(1\right)=0$, *V*_*i*_(ϵ) = 0 and $V_{i}^{{}^{\prime \prime }}\left(\epsilon \right)=0$. For ϵ ≤ 0.35, Equation (5) was solved numerically up to three conical eigenmodes using Mathematica 8.0 (Wolfram Research Inc., USA). For ϵ > 0.35 however, due to numerical instabilities, only the first two conical eigenmodes could be calculated. Following unpublished work by Svoboda and coworkers, a second method was thus implemented and consisted in decomposing the conical eigenmodes onto the eigenmodes of a cylindical rod having the same length *L*, same Young's modulus *E* and same mass density ρ as the conical one, and a radius taken as its base radius *b* (see Supplementary Material for a full derivation).
  Amplitudes *q*_*i*_ for each eigenmode *V*_*i*_ were computed following (Boubenec et al., 2012). One can show that *q*_*i*_ evolves with time *t* according to the following equation
  (6) $$q_{i}(t)=-A\left(\int_{\varepsilon }^{1}s^{2}\bar{U}(s)V_{i}(s)ds\right)G(t)$$
  with *A* = *V*(*resp*.*Lγ*) for the fixed whisker experiment (*resp*. rotating whisker experiment). In Equation (6), *Ū* is defined by the relationship *Ü*_*qs*_(*s, t*) = δ(*t*)*AŪ*(*s*) where Ü_*qs*_ is the second time-derivative of the quasi-static profile, δ(*t*) is the Dirac function, and *G*(*t*) is the Green's function for the resonant system given by
  (7) $$G(t)=\frac{e^{-\zeta \omega_{i}t}\text{sin}(\sqrt{1-\zeta^{2}}\omega_{i}t)}{\sqrt{1-\zeta^{2}}\omega_{i}}$$
  where ζ is a damping factor assumed to be constant. The integral in Equation (6) was computed numerically using Matlab 2015 (Mathworks Inc., USA).

**Table 2:** **Quasi-static deflections and related base torque for shock experiments**.

	**Fixed whisker**	**Rotating whisker**
Boundary conditions	*U*_*qs*_(1, *t*) = 0, $\frac{\partial U_{qs}\left(1,t\right)}{\partial s}=0$	*U*_*qs*_(1, *t*) = 0, $\frac{\partial U_{qs}\left(1,t\right)}{\partial s}=\gamma t$
	*U*_*qs*_(ϵ, *t*) = *Vt*,$\frac{\partial^{2}U_{qs}\left(\epsilon ,t\right)}{\partial s^{2}}=0$	*U*_*qs*_(ϵ, *t*) = 0,$\frac{\partial^{2}U_{qs}\left(\epsilon ,t\right)}{\partial s^{2}}=0$
*U*_*qs*_(*s, t*)	$Vt\frac{\varepsilon {\left(1-s\right)}^{2}\left(\varepsilon -3s+2s\varepsilon \right)}{2{\left(\varepsilon -1\right)}^{3}s^{2}}$	$L\gamma t\frac{\left(s-1\right)\left(2s^{2}+\varepsilon^{2}\left(1+s\right)-\varepsilon s\left(3+s\right)\right)}{2{\left(1-\varepsilon \right)}^{2}s^{2}}$
*M*_*qs*_(*t*)	$\frac{3E\pi b^{4}}{4}\;\left(\frac{1}{d^{2}}-\frac{\alpha }{d\;b}\right)\;Vt$	$\frac{3E\pi b^{4}}{4}\left(\frac{1}{d}-\frac{\alpha }{b}\right)\gamma t$

#### 2.3.2. Free whisking in air

In this case, the whisker is considered to be fixed at its base, while the base angle φ(*t*) is driven sinusoidally according to φ(*t*) = φ_0_ *sin*(2π*f*_*w*_*t*), where *f*_*w*_ is the whisking angular frequency. No additional constraints are applied to the rest of the whisker that can move freely in air.

- Quasi-static deflections
  Since the whisker is supposed to have no intrinsic curvature, quasi-static deflections *U*_*qs*_(*s, t*) are solid rotation-like and thus simply given by
  (8) $$U_{qs}(s,t)=L\varphi (t)(1-s)$$
- Dynamic deflections
  Because of the whisker's inertia in air, vibrations will propagate along the whisker for high angular velocities. Dynamic deflections can be decomposed onto the spatial eigenmodes *V*_*i*_(*s*). These are obtained by solving Equation (5) with the constraints *V*_*i*_(1) = 0, $V_{i}^{\prime }\left(1\right)=0$, $V_{i}^{{}^{\prime \prime }}\left(s_{tip}\right)=0$ and $V_{i}^{{}^{\prime \prime \prime }}\left(s_{tip}\right)=0$, as both the torque and the force are equal to zero at the whisker's tip (whose curvilinear coordinate is *s*_*tip*_, see Figure 2A). Equation (5) was solved numerically using Mathematica 8.0 (Wolfram Research, USA) to compute the first five eigenmodes and eigenfrequencies. As shown in Boubenec et al. (2012), the dynamic term is given by
  (9) $$u(s,t)=\sum_{i}V_{i}(s)\left(\int_{s_{tip}}^{1}s^{2}V_{i}(s)ds\int_{0}^{t}G(t-t^{\prime }){\ddot{U}}_{qs}(s,t^{\prime })dt^{\prime }\right)$$
  where Ü_*qs*_ is the second time derivative of *U*_*qs*_ computed from Equation (8) and given by
  (10) $${\ddot{U}}_{qs}(s,t)=-L\omega_{w}^{2}\varphi_{0}(1-s)\text{sin}(\omega_{w}t)$$
  Combining Equations (7), (9) and (10) allows separation of space and time variables, yielding the following final expression for *u*(*s, t*)
  (11) $$\begin{array}{l} u(s,t)=\frac{-L\omega_{w}^{2}\varphi_{0}}{\sqrt{1-\zeta^{2}}}\;\sum_{i}\frac{V_{i}(s)}{\omega_{i}}\left(\int_{s_{tip}}^{1}s^{2}V_{i}(s)(1-s)ds\right)\\ \times \int_{0}^{t}e^{-\zeta \omega_{i}(t-t^{\prime })}\\ \text{sin}\left(\sqrt{1-\zeta^{2}}\omega_{i}(t-t^{\prime })\right)\text{sin}(\omega_{w}t^{\prime })dt^{\prime } \end{array}$$
  The time integral in Equation (11) was computed using Mathematica 8.0.

## 3. Results

A first set of experiments involving simple whisker/object contacts at different shock velocities have been performed, whose results are reported in the following sections “Quasi-static regime” and “Dynamical regime.” The objective is to separately probe the quasi-static (low shock velocity) and vibrational (high shock velocity) components of the mechanical stresses elicited at the base of the whisker. Since the vibrational component of the base torque *M* cannot be directly measured with the rheometer, optical measurements of the base curvature *C* are used and *M* − *C* calibration procedure is first reported. We then continue this section by presenting how both quasi-static and dynamical components of the base torque vary with the radial distance *d* and by giving a detailed comparison of our measurements with the biomechanical model described earlier. To reproduce natural exploration tasks used by a rodent to detect and localize an object in its vicinity, a second set of experiments have also been performed and are reported in the section “Whisking regime.” These consisted in approaching an object to an oscillating whisker to mimic both the body motion and the whisking behavior. Both quasi-static and dynamical components of the torque at the base of the whisker are measured and confronted with the predictions of our biomechanical model.

### 3.1. Curvature—base torque calibration

**Figure 3:**
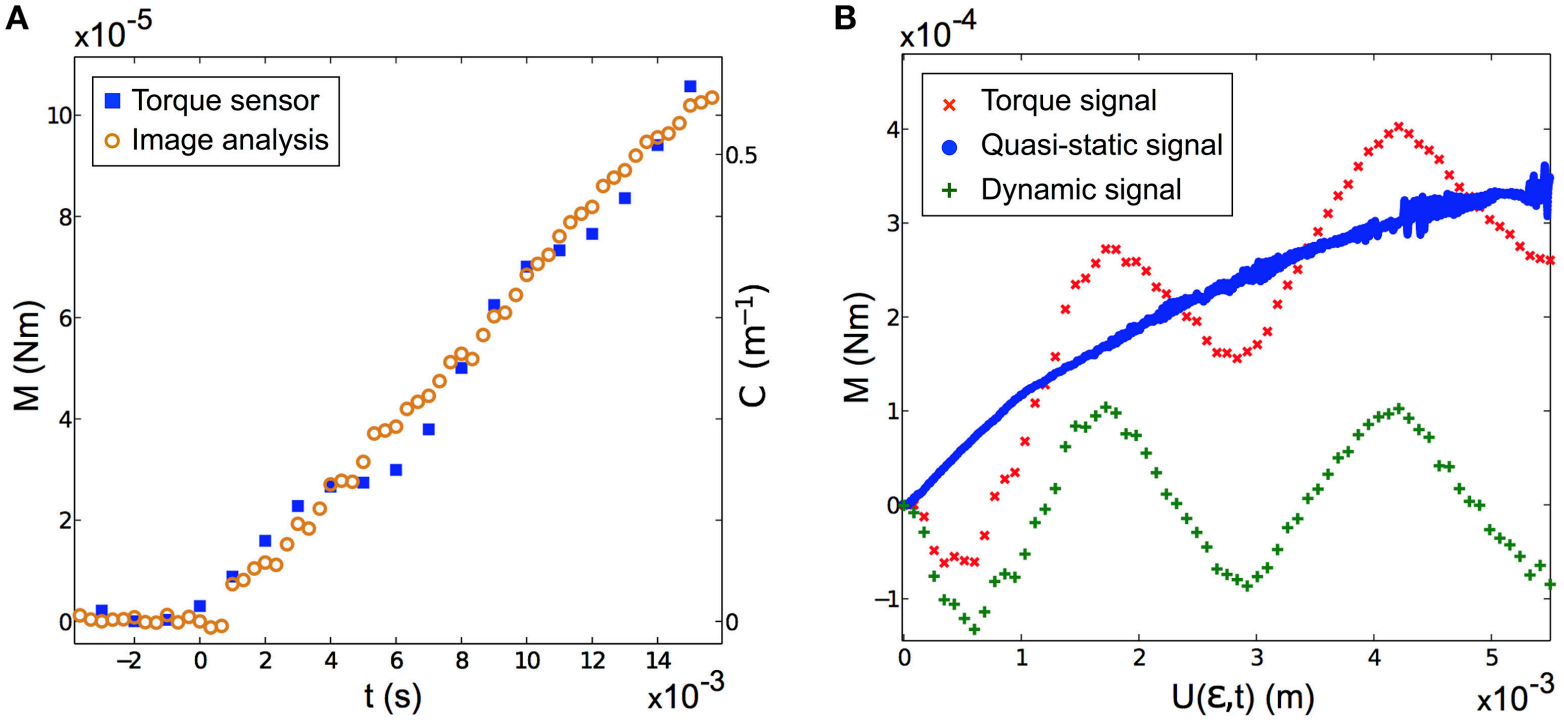
**(A)** Base torque *M*(*t*) measured by the rheometer (blue squares) and base curvature *C*(*t*) obtained by image analysis (orange circles) in a *fixed artificial whisker experiment* (*V* = 2.743 ± 0.002 cm s^−1^, ϵ = 0.49 ± 0.03, *f*_*cam*_ = 3000 fps). The proportionality factor between *M*(*t*) and *C*(*t*) is found to be *K* = (1.8 ± 0.1)10^−4^ Nm^2^. **(B)** Typical measured base torque signal vs. the contact point displacement *U*(ϵ, *t*), in a *fixed artificial whisker experiment* (ϵ = 0.45 ± 0.03). Shown with the red crosses (*resp*. blue disks) is the measured signal for a large shock velocity *V* = 86.0 ± 0.1 cm s^−1^ (*resp*. a small shock velocity *V* = 1.17 ± 0.01 cm s^−1^). The difference between both signals yields the dynamic component (green plus signs). For both experiments, *f*_*cam*_ = 10, 000 fps.

To extract the base torque from the measurements of the base curvature $C\left(t\right)=\partial^{2}U\left(s,t\right)/\partial s^{2}{|}_{s=1}$, we developed a calibration procedure. It consisted in measuring, in a *fixed whisker experiment*, the base torque in the quasi-static regime *M*_*qs*_(*t*), in a configuration where the signal is in the measurable range of the rheometer torque sensor, and comparing it to the optically measured *C*(*t*). A proportionality *M*_*qs*_(*t*) = *K C*(*t*) was systematically observed with *K* ≈ *Eπb*^4^/4, as predicted by Equation (2). This calibration was done for both the artificial (see an example in Figure 3A) and the real whiskers. This calibration procedure thus provides an optical measure of *M*(*t*), even in the dynamic regime for which no direct torque measurement is possible. As an example, we show in Figure 3B a typical shock experiment performed with the artificial whisker at low/high velocities. At high shock velocity, the total torque signal evolves similarly as its quasi-static component but comprises additional vibrations which are clearly captured using the optical measurement.

### 3.2. Quasi-static regime

#### 3.2.1. Experiments

**Figure 4:**
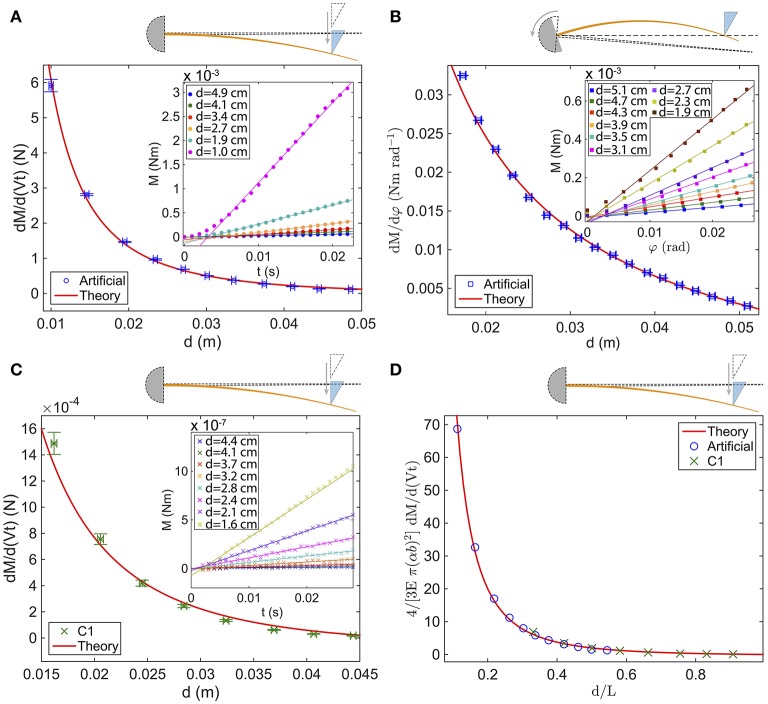
**Rate of change of the quasi-static base torque vs. ***d*** for (A)** the *fixed artificial whisker* (blue circles), **(B)** the *rotating artificial whisker* (blue squares) and **(C)** the *fixed real whisker* (green crosses). In **(A)** [*resp*. **(B)**], the inset shows the base torque measured with the rheometer vs. time *t* (*resp*. φ), for increasing *d*—top to bottom curves. Similarly, in **(C)** the inset shows the base torque obtained by image analysis vs. *t* for increasing *d*. **(D)** Dimensionless curve $4/\left(3E\pi {\left(\alpha b\right)}^{2}\right)dM_{qs}/d\left(Vt\right)$ vs. *d*/*L* for the *fixed artificial whisker* (blue circles) and the *fixed real whisker* (green crosses). In **(A–C)**, the red solid lines are fits with predicted rates of change (see Table 2). In **(A)**, *E* = 1.19 GPa, *V* = 2.7 cm s^−1^ and the fit yields [α = 7.8 ± 1.7 mrad, *b* = 700 ± 9 μm]. In **(B)**, *E* = 1.19 GPa and the fit yields [α = 11.3 ± 0.3mrad, *b* = 712 ± 5μm]. In **(C)**, α = 1.8 mrad, *b* = 88 μm, *V* = 2.7 cm.s^−1^ and the fit yields *E* = 3.6 ± 0.4 GPa. In **(D)**, the red solid line represents the function *f*(x) = 1/x^2^−1/x. Error bars represent the standard deviation.

Experiments are performed at low indenter velocities, so that the torque depends solely on the indentation, regardless of the amplitude of the chosen velocity. This is done both with a fixed whisker and a moving object (Figures 4A,C) and a rotating whisker and fixed object (Figure 4B). Base torque signals *M*_*qs*_ are shown in the insets of Figure 4 for both artificial (Figures 4A,B) and real whiskers (Figure 4C). All of them show that *M*_*qs*_ increases linearly with indentation *Vt* (*resp*. with φ for the case of the rotating whisker), for all probed radial distances *d*. For each *d*, *M*_*qs*_ is fitted linearly, yielding a rate of change *dM*_*qs*_/*d*(*Vt*) (*resp*. *dM*_*qs*_/*dφ*) that varies monotonically with *d* (see the main panels of Figures 4A–C). Note that the closer the contact point from the base, the larger *dM*_*qs*_/*d*(*Vt*) (*resp dM*_*qs*_/*dφ*).

#### 3.2.2. Comparison to the model's predictions

Predictions for *dM*_*qs*_/*d*(*Vt*) (*resp*. *dM*_*qs*_/*dφ*), obtained by deriving with respect to time (*resp*. φ) the analytical expressions of *M*_*qs*_ given in Table 2, were fitted to the experimental data points. For the artificial whisker, *E* and *V* have been measured accurately, and α and *b* are thus the only fitting parameters. For the real whisker, since *E* was not measured, we chose to leave it as a fitting parameter, while imposing α, *V* and *b*. Results are shown with the red solid lines on Figures 4A–C. In all cases, a very good agreement between the theoretical predictions and the experiments is found. For the fixed (*resp*. rotating) artificial whisker experiment, the fits yield α = 7.8 ± 1.7 mrad and *b* = 700 ± 9 μm (*resp*. α = 11.3 ± 0.3mrad and *b* = 712 ± 5 μm), close to the values measured directly (see Table 1). For the real whisker experiment, the value of *E* = 3.6 GPa resulting from the fit also falls within the range of reported values for rat whiskers (Quist et al., 2011). Interestingly, the data of Figures 4A,C fall on the same master curve (Figure 4D), provided that the abscissa axis is divided by *L* and the ordinate one is multiplied by 4/(3*VEπ*(α*b*)^2^), since

(12) $$\frac{4}{3E\pi {\left(\alpha b\right)}^{2}}\;\frac{dM_{qs}(t)}{d(Vt)}=\frac{1}{{\text{x}}^{2}}-\frac{1}{\text{x}}$$

with x = *d*/*L*. Such a result demonstrates that the mechanical properties of the real whisker are faithfully mimicked by our artificial whisker.

### 3.3. Dynamical regime

#### 3.3.1. Wave front propagation

**Figure 5:**
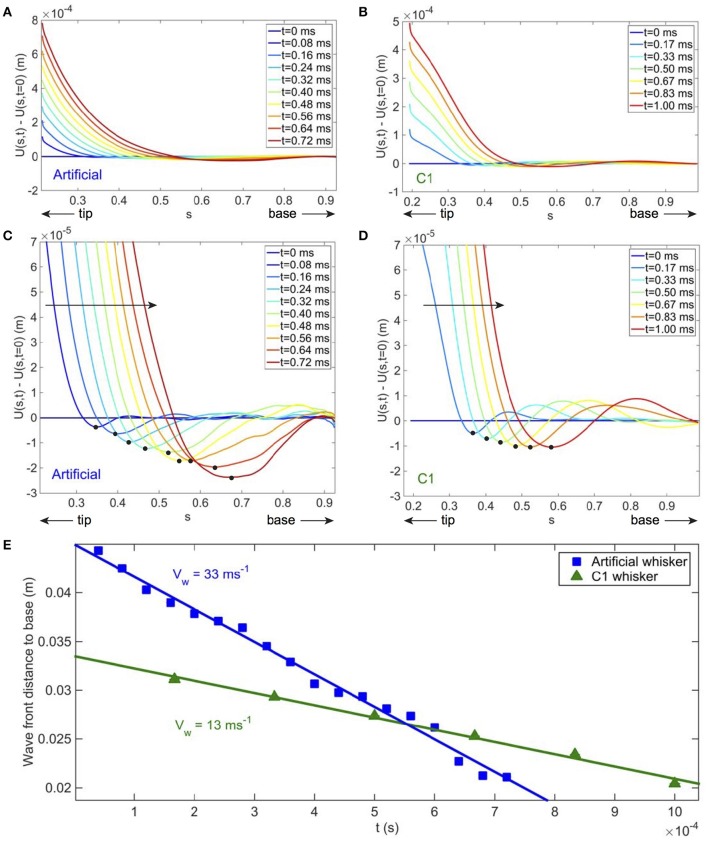
**Wave front propagation after a shock in ***fixed whisker experiments***. (A)** [*resp*. **(B)**] Total artificial (*resp*. real) whisker displacements vs. *s* at increasing times *t*—blue to red colors (*V* = 99 ± 3 cms^−1^). For the artificial whisker in **(A)** ϵ = 0.21 ± 0.01, *f*_*cam*_ = 25, 000 fps; for the real whisker in **(B)** ϵ = 0.19 ± 0.01, *f*_*cam*_ = 6000 fps. Whiskers profiles are smoothed on a 41-pixel (~ 3 mm) wide window. **(C,D)** Close up of **(A,B)** to visualize the deflection wave. On both **(C,D)**, the large solid arrow indicates the direction of propagation of the wave and the black disks localize the wave front position. **(E)** Wave front distance to base vs. *t* for the artificial whisker (blue solid squares) and the real one (green solid triangles). Fitting linearly the data points yields a wave propagation velocity *V*_*w*_ = 33 ± 2 m s^−1^ and *V*_*w*_ = 13 ± 1 m s^−1^ for the artificial and real whiskers, respectively. Error bars are smaller than the size of the symbols used.

At high shock velocity of the indenter, a deflection wave is triggered at the contact point and propagates toward the base. Such deflections can be best identified by subtracting to the whisker profile at time *t* its profile at the time of the shock. Figures 5A,B and their associated close-ups (Figures 5C,D) show such deflections at different instants for the artificial and real whisker, respectively. Note that the curvature of the induced deflections has an opposite sign to that of the quasi-static one. The position of the wave front, defined as the position of the minimal deflection (and shown with the black disks on Figures 5C,D), is presented on Figure 5E as a function of time for both artificial and real whiskers. The wave fronts propagate at a constant velocity as theoretically predicted in Boubenec et al. (2012). For a cylindrical whisker, one would expect $V_{\text{w}}\sim \sqrt{t}$ (Audoly and Neukirch, 2005). A linear fit yields a wave front velocity *V*_w_ = 33 ± 2 m.s^−1^ for the artificial whisker, and *V*_w_ = 13 ± 1 m.s^−1^ for the real whisker. Using dimensional analysis, this wave velocity is expected to be proportional to $\alpha \sqrt{E/\rho }$, where $\sqrt{E/\rho }$ is the speed of sound within the bulk material and α the conical angle. With the parameters of Table 1, the ratio of wave velocities *V*_*w*_ for the artificial to the real whiskers is thus expected to be 2.7. Experimentally, this ratio is 2.5 ± 0.3.

#### 3.3.2. Dynamic signature in the base torque

**Figure 6:**
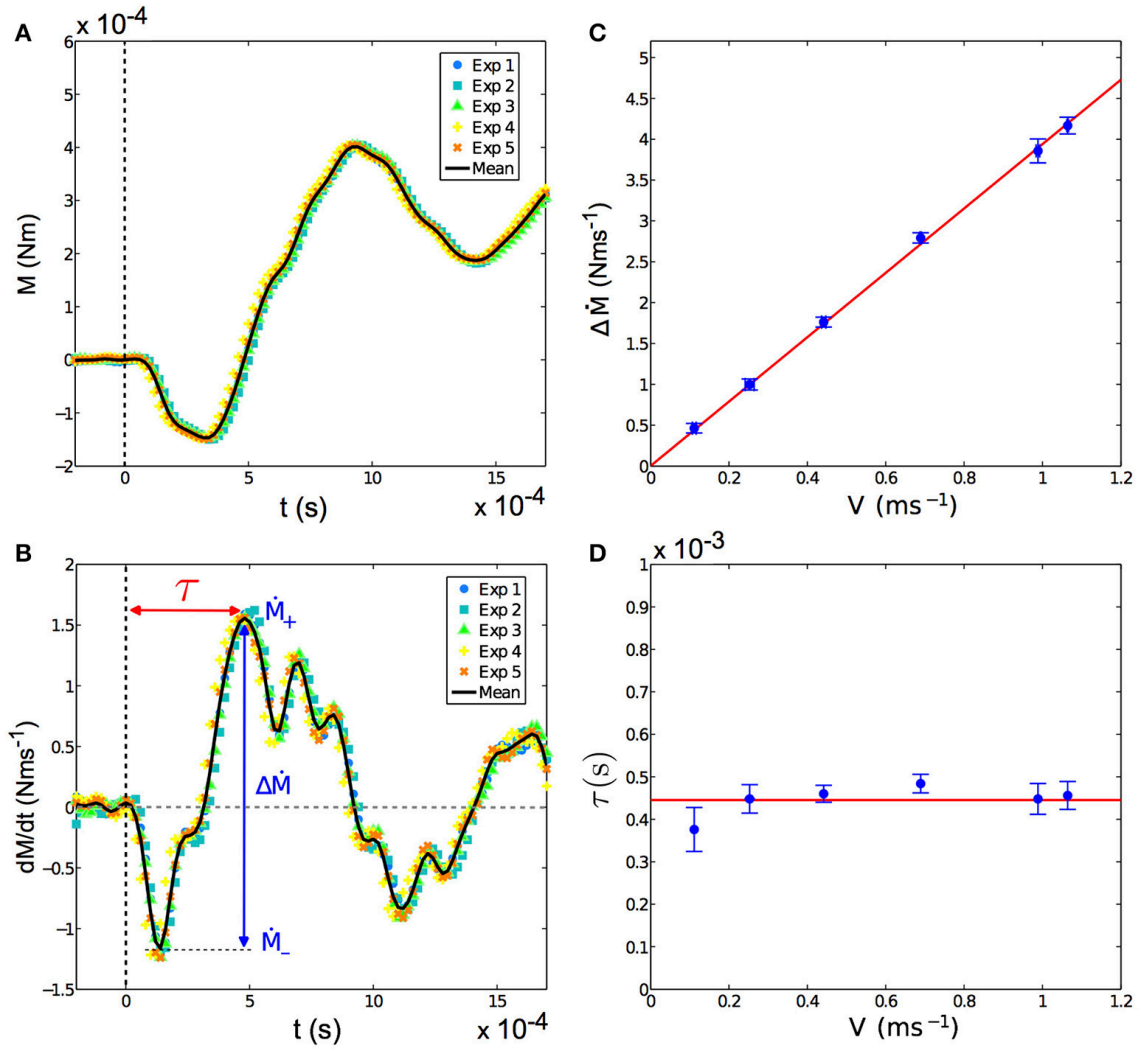
**(A)** Torque at the base of the artificial whisker vs. time *t*, in a *fixed whisker experiment*, immediately after the shock (taken at *t* = 0) for five repeated shocks in the same experimental conditions (colored symbols), namely ϵ = 0.44 ± 0.02 and *V* = 68.9 ± 0.2 cm s^−1^. The solid black line is the mean torque averaged over all five experiments. **(B)** Time derivative of the base torque shown in **(A)** vs. *t*. The shock triggers at the base a large oscillation with a maximal variation Δ*Ṁ* that is reached after a time delay τ. **(C)** Amplitude Δ*Ṁ* vs. shock velocity *V*. The red solid line is a linear fit of the data of the form *pV* with *p* = 3.94 ± 0.07 N. **(D)** Delay τ vs. *V*. The red solid line corresponds to the mean value of τ averaged over all τ(*V*), equal to (4.5 ± 0.4)10^−4^ s. In both **(C,D)**, ϵ = 0.44 ± 0.02 and *f*_*cam*_ = 25, 000 fps. Error bars represent the standard deviation.

The propagation of this deflection wave front from the contact point down to the base induces a specific signature in the base torque at short times (*t* < 10 ms). Such a signature can be seen, for the case of the artificial whisker, on Figure 6A. Immediately after the shock (which occurs at *t* = 0), the incoming wave front induces a negative curvature (and thus a negative base torque) with respect to the quasi-static one. This characteristic decrease in the base torque occurs a few milliseconds after the shock and could be used by rodents to detect the instant of the shock. This slight decrease of the base torque is followed by positive oscillations which damp out over time (Figure 6A). To quantify the dynamic component, we have computed the time derivative of the base torque *dM*/*dt*. As shown on Figure 6B, the latter exhibits large oscillations immediately after the shock with a maximum amplitude Δ*Ṁ* after a time delay τ. We have measured how the exploratory conditions (shock velocity and radial position of the indenter with respect to the base) affect the amplitude of Δ*Ṁ* and the time delay τ. We present on Figure 6C the variations of Δ*Ṁ* with the shock velocity *V*, for a fixed contact point. A linear relationship is observed, in agreement with the prediction of the model. The mode amplitude *q*_*i*_ given by Equation (6) is indeed expected to be proportional to *V*. As a consequence, the model predicts that the dynamical contribution of the base torque and Δ*Ṁ* are also proportional to *V*. On the contrary, the delay τ is expected to be independent of *V* as it arises from the propagation of deflections from the contact point to the base. As long as the contact point is fixed, which is the case here, we thus expect τ to depend only on geometrical and mechanical properties of the whisker but not on the shock velocity. Experimentally, we indeed observe that τ does not show significant variations with *V*, as evidenced in Figure 6D.

**Figure 7:**
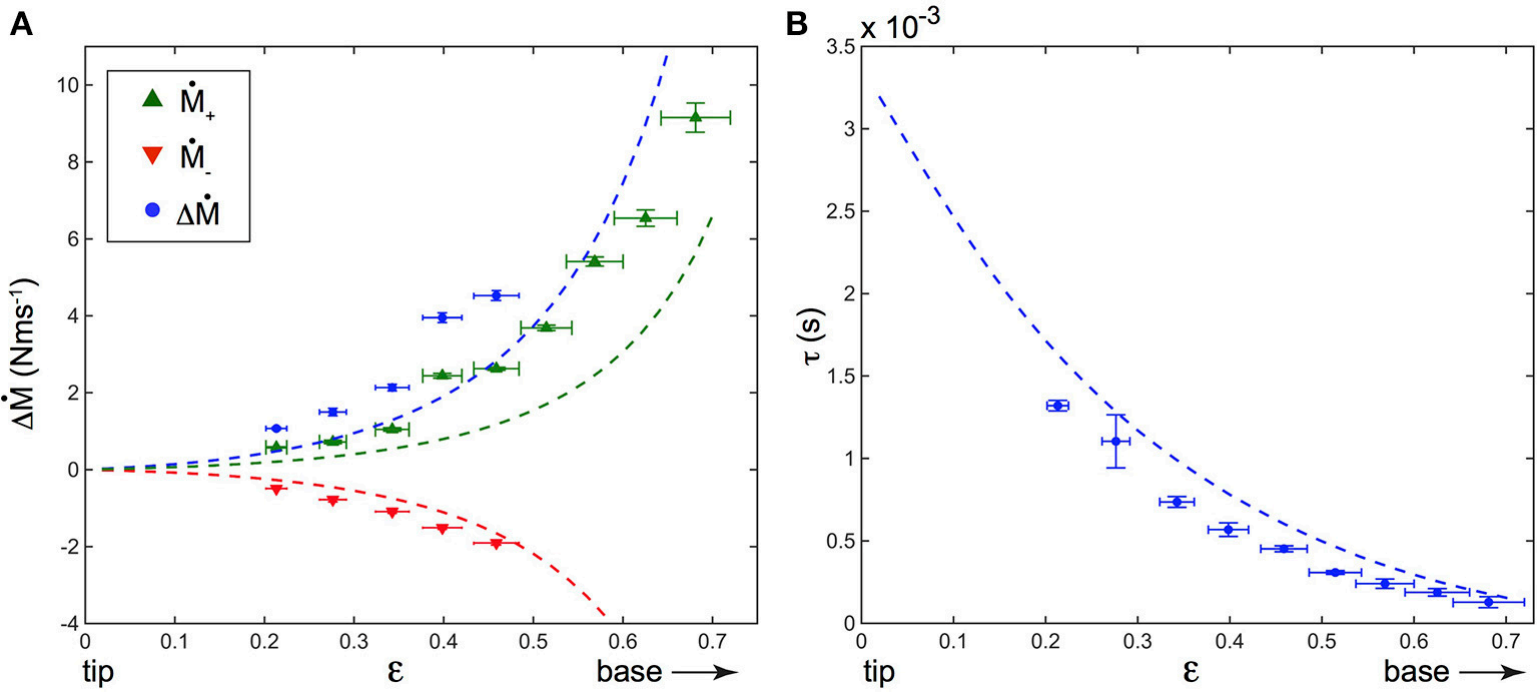
**(A)** Dependence of Δ*Ṁ* = *Ṁ*_+_−*Ṁ*_−_ (blue disks) with the contact point location ϵ in a *fixed artificial whisker experiment*, along with that of *Ṁ*_+_ (green upward triangles) and *Ṁ*_−_ (red downward triangles). **(B)** τ vs. ϵ (blue disks). On both **(A,B)**, *V* = 99 ± 3 cm s^−1^, *f*_*cam*_ = 25, 000 fps, and the dashed lines correspond to the predictions of the model. Error bars represent the standard deviation.

For a constant shock velocity, Δ*Ṁ* is found to increase with the contact point location ϵ, meaning that the closer the contact point to the base, the larger the dynamical part of the base torque (Figure 7A). The delay τ also decreases with ϵ (Figure 7B), being smaller when the contact is closer to the base. This is to be expected since the wave propagates at constant velocity *V*_*w*_, and thus the time delay for a deformation to reach the base will decrease with increasing ϵ.

#### 3.3.3. Comparison to the model's predictions

The shock induced deflections of the whisker predicted by the model were computed using Equations (5), (6) and (7). The amplitudes *q*_*i*_(*t*) were obtained by numerically integrating Equation (6). The value of the damping ratio ζ was determined experimentally during a detachment process, for which an initially constrained whisker relaxes freely in air. The base torque was found to decay pseudo-periodically with time *t* and was well fitted (not shown) with $M\left(t\right)=\;A\;e^{-\zeta \omega t}\;\cos\left(\sqrt{1-\zeta^{2}}\omega t\right)$. This fit yielded a damping ratio ζ = 0.041 ± 0.002, which was further assumed to be independent on ϵ (Boubenec et al., 2012). Base torques *M* were then deduced from the computed displacement profiles using Equation (2). Finally, both Δ*Ṁ* and τ were evaluated for different ϵ. Theoretical predictions for both Δ*Ṁ* and τ dependence with ϵ are plotted on Figure 7 and compared to the experimental measurements. As it can be seen, the model is in good agreement with the experimental data. Without any adjustable fit parameters, the values and time delays of the dynamical component of the base torque are correctly captured as well as their dependence with the contact point location. As for the quasi-static component, the dynamic counterpart shows a monotonic increase of Δ*Ṁ* when the radial distance *d* decreases.

### 3.4. Whisking regime

**Figure 8:**
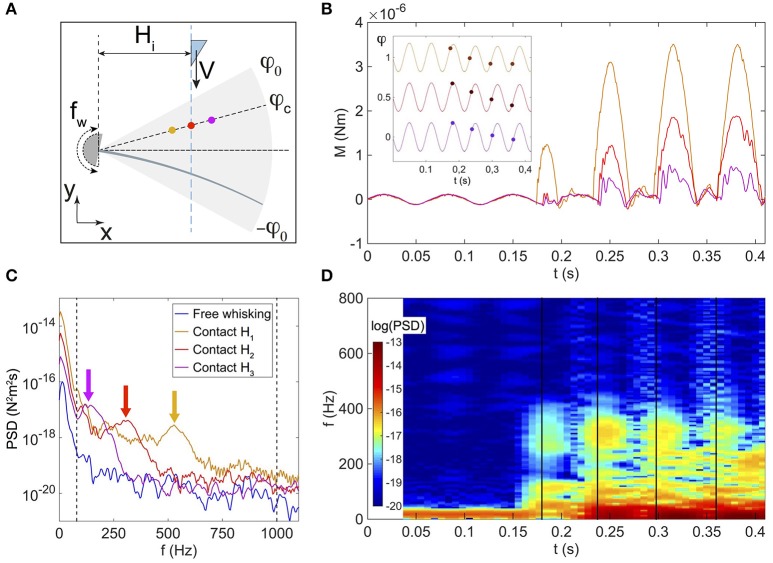
**(A)** Sketch of the *whisking experiment*. Horizontal distances *H*_*i*_ separating the whisker base and trajectory of the object are fixed and assume three values *H*_1_ = 2.07 ± 0.01 cm (orange color), *H*_2_ = 2.60 ± 0.01 cm (red color), and *H*_3_ = 3.17 ± 0.01 cm (magenta color). The angular amplitude of the whisking is φ_0_, and the position of the object is defined by both the angle φ_*c*_ at contact and the distance *H*. **(B)** Base torque *M* as a function of time *t* for the three *H* values as sketched in **(A)**. Inset: whisker base angle as a function of *t*. For sake of clarity, curves have been arbitrarily shifted vertically. Disk symbols mark the instants of contact with the object and corresponding φ = φ_*c*_ values. **(C)** Power Spectral Density (PSD) plot of the base torque signals averaged on all contacts, and performed over a duration of 14.7 ms from the time of contact. The blue solid line represents the base torque PSD averaged over the free whisking phases. The two vertical black dashed lines are drawn at the low and high cutoff frequencies, respectively 80 and 1000 Hz. The arrows indicate the vibration frequencies *f*_0_. **(D)** Spectrogram (in logarithmic scale) of *M* for the experiment performed at *H*_2_. Vertical black solid lines mark the instants of contact.

We used a biomimetic approach to reproduce (in a crude approximation) the typical pattern of exploration of nearby objects adopted by rodents, taking into account both body and vibrissae *whisking* motions (for details, see the Section entitled “Materials and Methods” and a movie of the experiment in the Supplementary Material). The same wedge is now moved toward an oscillating whisker, along a straight trajectory parallel to the *y* axis at a horizontal distance *x* = *H*_*i*_ with the index *i* = {1, 2, 3} standing for the three separation distances reported here (see Figure 8A). The object is then abruptly stopped at a prescribed *y* position, and the whisking motion of the whisker against the fixed object pertains, inducing successive identical shocks. For the sake of simplicity, the body/object dynamics is restricted to a 1D translation at constant velocity and the whisking is purely sinusoidal.

The whisking is simulated by forcing the base angle of the whisker to oscillate in air at a frequency *f*_w_=15 Hz and with an amplitude φ_0_=10°, using the rheometer head in a sinusoidal mode (Malkin and Isayev, 2006). For these experiments, the real whisker has been used rather than the artificial one, for reasons that will appear clear further down. During both *free whisking in air* and *contact* phases, the deflections of the whisker are tracked with the fast camera, allowing to extract the base torque as described earlier.

Figure 8B shows the time evolution of the base torque. Due to the inertia of the whisker, whisking in air (which occurs at times *t* < 0.18 s on Figure 8B) induces modulations of the base torque around the whisking frequency *f*_w_, with a typical amplitude $\sigma_{\text{w}}\sim 10^{-7}$ Nm. Note that with the artificial whisker, inertia induced deflections were too small to be measured, which explains why our experiments were solely performed with the real whisker. As the object enters the spatial region swept by the whisker during a whisking cycle, successive contacts occur. For any given contact, the base torque displays the typical shock-induced shape described in the previous sections, and consisting of a quasi-static linear increase with superimposed vibrations. We have plotted on Figure 8C the Power Spectral Density (PSD) of the base torque signal, performed over a duration of 14.7 ms from the time of contact, and averaged over 5 repetitions of the same experiment. These PSD plots display two main peaks, a first one at the whisking frequency *f*_*w*_ and a second one at a higher frequency *f*_0_ whose value decreases with the radial position *d* and is related to the typical vibration resonance frequency of the whisker. Shown on Figure 8D is a spectrogram for the example of the experiment done at the horizontal distance *H*_2_, with the same time axis as in Figure 8B. Here again, the free whisking phase in air is characterized by a maximum of the PSD at low frequencies around *f*_*w*_, while all successive contacts trigger high frequency signals as evidenced by the local maxima of the PSD around 300 Hz. Such behavior is also observed for the two other experiments performed at *H*_1_ and *H*_3_. Interestingly, in all cases, both frequencies are well apart from each other, with shock induced vibrations having high frequencies contents above 100 Hz, well above the whisking frequency.

In the context of the detection of contacts by rodents, it is important to notice that since the base torque value oscillates during free whisking in air, the *first contact* might be *undetected* using the total torque signal. For example, looking at the torque signal for the experiment at horizontal distance *H*_2_ (red curve on Figure 8B) the first contact occurs at a time *t* ≈ 0.18 s when φ_*c*_ is close to its maximum value, i.e., at the end of the protraction. The first contact induces a limited increase of the base torque, with a maximum amplitude that remains lower than σ_w_. In contrast, the two subsequent contacts (*t* > 0.23 s on Figure 8B) occur while the object is still moving toward its stop position and for both of them sooner in the protraction cycle. Both are characterized by a base torque which overpasses σ_w_.

**Figure 9:**
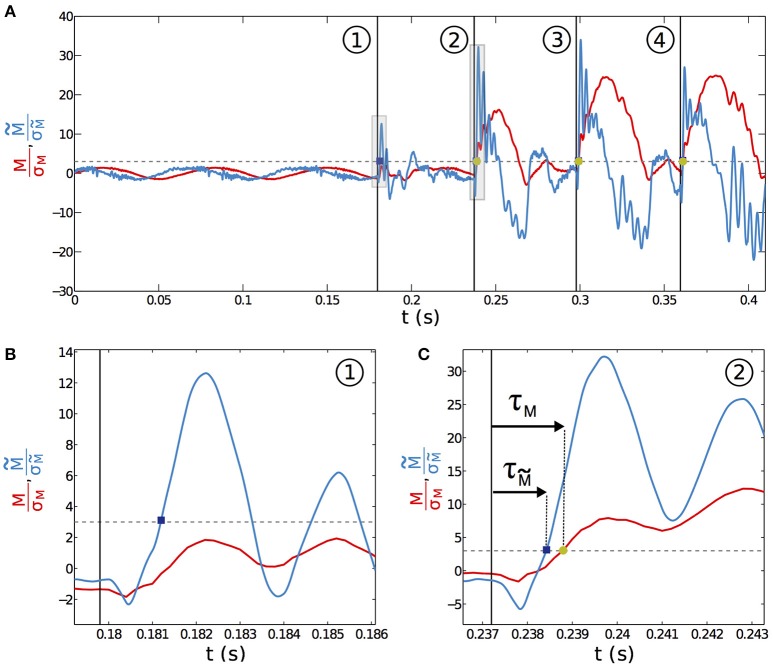
**Acuity and delay of contact detection. (A)** Normalized base torque *M*/σ_*M*_ for the whisking experiment (red solid line) with 4 successive contacts labeled ①, ②, ③ and ④. The normalized filtered base torque $\tilde{M}/\sigma_{\tilde{M}}$ (blue curve) is obtained by applying a first order bandpass filter to the total base torque signal. The vertical lines mark the onset of contact. The horizontal dashed line represents *M* = 3σ_*M*_. **(B)** Close up at contact ① in **(A)** (shaded area). **(C)** Same with contact ②. The time delay between the onset of contact and the time at which *M* > 3σ_*M*_, (resp. $\tilde{M}>3\sigma_{\tilde{M}}$) is denoted τ_*M*_ (resp. $\tau_{\tilde{M}}$).

It seems reasonable to hypothesize that σ_w_ sets a typical mechanical threshold that has to be exceeded for a contact to be detected using the total base torque signal. The base torque plotted on Figure 9A is normalized by σ_w_. To be less sensitive to noise, we chose the following arbitrary convention. A contact will be detected if *M*/σ_w_ > 3 (as shown by the dashed line on Figure 9A). With this convention, the first contact (labeled with the circled number ① on Figure 9A) is therefore not detected using the total base torque signal. On the contrary, both subsequent contacts do exceed the chosen threshold and are therefore detected. With this simple example, we already capture an important feature that will be discussed below in more details. Detection of contacts based on the torque signal is sensitive to the exploratory conditions, such as the contact point location and the protraction angle φ_*c*_ at which contact occurs.

**Figure 10:**
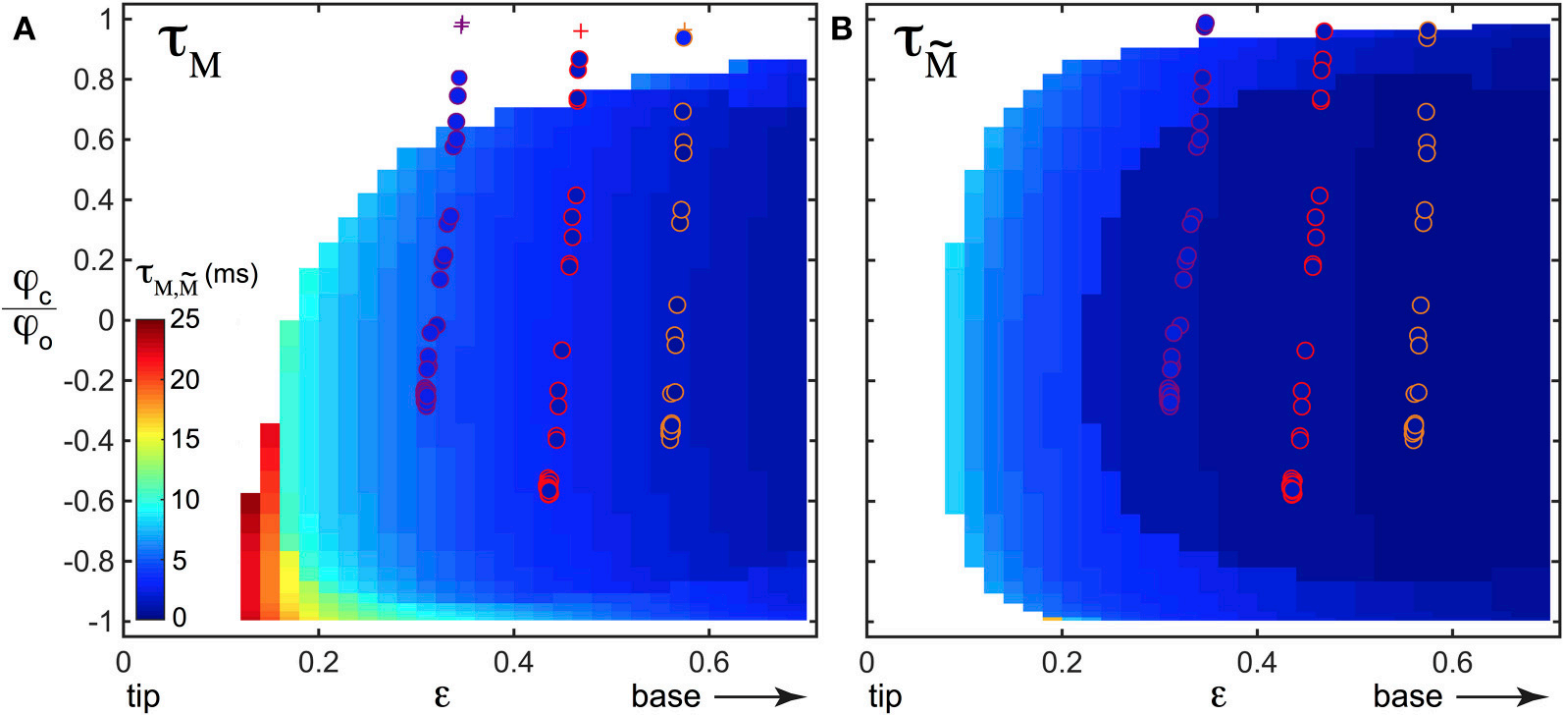
**Colormaps of (A)** τ_*M*_(ϵ, φ_*c*_/φ_0_) and **(B)** $\tau_{\tilde{M}}\left(\epsilon ,\varphi_{c}/\varphi_{0}\right)$ obtained with numerics. The white zones correspond to undetected contacts. Detected contacts are shown with the color bar whose units are given in milliseconds. On both **(A,B)**, data points of all three experiments performed at *H*_1_, *H*_2_, and *H*_3_ have also been plotted for comparison, with disk symbols when the contact is detected and plus sign symbols when it is not. The colors of the edge of the disks and of the plus signs have been chosen to match those of Figures 8A–C and correspond to different *H*_*i*_ [*H*_1_ (orange color), *H*_2_ (red color), *H*_3_ (magenta color)]. The inner color of the disks gives the value of the detection time, using the same color code as the one used to represent the numerical results.

A detection strategy based on the slowly time varying signal of the base torque can fail in detecting all contacts in our biomimetic whisking experiment. What about the fast shock induced vibrations? To isolate the dynamical component and since whisking and vibration frequencies are decoupled, we filtered the base torque measurements with a first order bandpass filter [frequency range (80–1000) Hz, centered on the typical vibration frequencies]. The filtered base torque signal $\tilde{M}$ is plotted on Figure 9A (blue curve and normalized as described below). During whisking in air, the filtered signal displays oscillations, mainly at the whisking frequency *f*_w_, with a mean amplitude $\sigma_{\tilde{M}}$. When the first contact occurs, the filtered signal shows a drastic increase. The shock induced vibration frequency falls within the bandwidth of the filter whereas *f*_w_ does not. The amplitude of $\tilde{M}$ is therefore much larger than $\sigma_{\tilde{M}}$. In contrast with the detection mode based on the total torque, the filtered signal allows detection of every contact in this experiment. As we will show below, this dynamical detection mode is indeed less sensitive to the exploratory conditions. This better detection of the first contact by the filtered dynamical signal has systematically been observed in many experimental trials. A good parameter to characterize the sensitivity to the exploratory conditions must be the time required from the onset of contact, to overpass the threshold of contact detection. We denote τ_*M*_ this time to have *M* > 3σ_w_ and $\tau_{\tilde{M}}$ its equivalent to have $\tilde{M}>3\sigma_{\tilde{M}}$ (see Figures 9B,C). To extensively cover the phase space [ϵ–φ_*c*_] we have simulated the whisking behavior, both in air and in contact with a fixed object, occurring at *s* = ϵ and for a given angle φ_*c*_ normalized by the whisking amplitude φ_0_. The value φ_*c*_/φ_0_ = −1 (*resp*. = 1) means that the contact occurs at the very beginning (*resp*. end) of the protraction. As shown in the Materials and Methods Section, σ_w_ can be computed theoretically by considering a whisker oscillating in air. As in the experiment, we chose *f*_w_ = 15 Hz and φ_0_ = 10°. Then, using the quasi-static and dynamic solutions of the model presented in this paper, we computed the total base torque *M*, as well as the filtered base torque $\tilde{M}$, using the same bandpass filter. We could thus compute σ_w_ and τ_*M*_ on one hand and $\sigma_{\tilde{M}}$ and $\tau_{\tilde{M}}$ on the other hand, for a large range of [ϵ–φ_*c*_]. The corresponding color maps τ_*M*_(ϵ, φ_*c*_) (*resp*. $\tau_{\tilde{M}}\left(\epsilon ,\varphi_{c}\right)$) are plotted on Figure 10A (*resp*. 10B). Both of them contain the features observed in the experiment of Figure 9A. When the contact occurs close to the tip, or at the end of the protraction cycle (such as the first contact of the experiment), it may not be detected by using the total torque signal. The number of undetected contacts (white zones in Figure 10) is larger in the case of the τ_*M*_ map than in the case of the $\tau_{\tilde{M}}$ map. According to these maps, the contact labeled ① is not detected using the torque signal (red plus sign symbol on Figure 10A), but it is with the filtered torque signal (most upper red circle on Figure 10B). In addition, the dynamical detection gives an almost flat distribution of detection times with respect to the exploratory conditions, in contrast with the total torque based detection method.

## 4. Discussion

Mechanical stresses acting on the base are the effective stimulation inputs for the sensory system. Our work provides a full determination of the whiskers deflections and induced base torque as whiskers encounter a sharp object. Measurements of the base torque for various radial positions of the object have been performed and confronted with theoretical predictions. Both slow (quasi-static) and rapid (vibrational) deflections have been characterized. We also developed a biomimetic whisking experiment and explored the robustness of detection using both mechanical modes. We discuss in the following consequences of these results.

### 4.1. Quasi-static deflections

Quasi-static deflections and induced base torques *M*_*qs*_ have been measured for artificial/real whiskers contacting a point-like object at different radial positions *d*. Since the quasi-static base torque is proportional to the indentation *Vt* (*resp*. φ) for the fixed (*resp*. rotating) whisker experiment, we have considered the rate of change of the base torque *dM*_*qs*_/*d*(*Vt*) (*resp*. *dM*_*qs*_/*dφ*) and its dependence on *d*.

Three points need to be highlighted. First, the rate of change of the quasi-static base torque has already been predicted theoretically in previous works (Birdwell et al., 2007; Solomon and Hartmann, 2008, 2011). Experimentally, Hartmann et al. have performed measurements on different types of rat whiskers (Birdwell et al., 2007), but for a limited number of *d* (and *d* < *L*/2). Here, we provide measurements on the *full range* of radial distances for both artificial/real C1 whiskers, and confront them to the theoretical predictions.

Second, all rates of change of the base torque for both artificial/real whiskers fall on a single master curve, when the dimensionless rate of change (4/(3*Eπ*(α*b*)^2^)*dM*/*d*(*Vt*)) is plotted vs. the dimensionless distance *d*/*L* (Figure 4D). Such a rescaling indicates that the whiskers mechanical properties can be - in a good approximation - modeled by an elastic truncated cone with a uniform Young's modulus. Furthermore, theoretical predictions of the rate of change of the quasi-static base torque (see Table 2) provide a very good fit to the data, using here again, a *constant* Young's modulus. The variation of *E* along the whisker length measured in Quist et al. (2011, 2014) might thus be a second order effect on the quasi-static mechanical properties.

Third, the rate of change of the quasi-static base torque is a monotonic—decreasing—function of *d*, and diverges when *d* approaches zero (Figure 4). It can therefore be used to locate radially an object as shown and discussed in Solomon and Hartmann (2011). Interestingly also, Table 2 shows that the quasi-static base torque increases linearly with the protraction angle φ with a slope inversely proportional to *d*. Since the base torque is itself proportional to the base curvature *C* (Equation (2)), it simply follows that

(13) $$C=3\left(\frac{1}{d}-\frac{\alpha }{b}\right)\varphi$$

Bagdasarian et al. (2013) have recently proposed that the phase space [*C*–φ] was appropriate and allowed localizing radially an object. Equation (13) rationalizes their observations.

### 4.2. Dynamic deflections

The whisker/object contact elicits the propagation of a transverse deformation wave from the contact point down to the base, better evidenced at high shock velocities *V*. We have measured for various *d* the induced base torque in this dynamical regime. A few milliseconds after the shock, the base torque displays characteristic modulations (Figure 6) better evidenced when looking at its time derivative *dM*/*dt* to disentangle quasi-static and dynamic components. The rate of change *dM*/*dt* displays modulations with a typical amplitude Δ*Ṁ* reached after a delay τ after the time of contact. Our results are in good agreement with the theoretical model we proposed (Boubenec et al., 2012) and recalled in the Materials and Methods section. First, we found that Δ*Ṁ* is proportional to *V*, whereas τ is independent of *V*, in excellent agreement with the model. This contrasts with the quasi-static signal, which is only proportional to the indentation amplitude regardless of *V* (Quist et al., 2014; Hartmann, 2015). Second, we show that Δ*Ṁ* (*resp*. τ) is a decreasing (*resp*. increasing) function of *d*, in good agreement with the model of Boubenec et al. (2012). Consequently, the dynamical signal could also be used to determine the radial localization of an object. Last, since τ is independent of *V*, the time delay of the dynamical signal arriving at the base is expected to be robust with respect to exploratory conditions.

The artificial system described in this study constituted an idealized approximation of the rat's tactile appendice. Hence, real whiskers are constantly growing at ~1 mm/day, resulting in a modification of their intrinsic resonant properties. However, the conical angle of the growing whiskers is relatively constant over time, such that the description of the shock-induced dynamics should be essentially independent of the whisker maturity. Another aspect is the mechanical anchorage of the whisker within the follicle. In the present study, we assumed a constant rigid attachment. A more realistic description would enable partial deformation of the follicle, with a torsional modulus that depends on blood volume in the sinus, or on the presence of another newly growing whisker. Such a modification could be implemented in experiments by coupling the artificial whisker and the force sensor with a rubber-like intermediate material. It could be accounted for in the model by assuming an elastic rather than a rigid boundary condition. Based on Boubenec et al. (2012), we expect such a refined model to yield rather similar phenomenology.

Last, let us note that we have only considered a single whisker. Multiple whiskers interactions can be observed during texture palpation (Ritt et al., 2008) and during facial interactions in rodents (Wolfe et al., 2011), but are beyond the scope of the present study

### 4.3. Quasi-static or dynamic?

In real settings, freely behaving rodents locate nearby objects with much more complex motor patterns than the ones reproduced with our simplified shock experiments. Indeed, in addition to body movements and head reorientations, a localization task involves a *whisking* behavior which consists in active and periodic protraction/retraction cycles of the whisker pad. A typical exploration sequence thus involves a first phase of free whisking in air while the animal is moving toward the object, followed by series of successive whiskers/object contacts. Such excitation mechanisms have been tested in behavioral experiments (McDonald et al., 2015). We have mimicked this exploration task with a sinusoidal oscillation of the whisker base as whisking and an object moving at constant velocity. Our results show that due to inertia of the whisker, the whisking in air induces modulations of the base torque at the whisking frequency *f*_*w*_. We hypothesize that these modulations of typical amplitude σ_*M*_ define a detection threshold using the total base torque to detect the contact. In contrast, the dynamical signal is excited at higher frequencies *f*_0_ whose typical values are well decoupled from *f*_*w*_. Due to this frequency decoupling, we isolated the dynamical component using a bandpass filter centered around *f*_0_. In addition, such decoupling induces a lower filtered signal in air (defining a lower threshold of typical amplitude $\sigma_{\tilde{M}}$) than the contact vibrations, favoring contact detection. Last, using numerics, we have shown that the dynamical detection mode is more robust to exploratory conditions (contact angle, radial position of the object). Our results suggest that first contacts are better detected with the dynamical mechanical signal.

The idea of defining a mechanical threshold from the whisking in air is supported by electrophysiological measurements (Leiser and Moxon, 2007). By recording the activity of Trigeminal Ganglion (TG) neurons of awake rats during *natural* whisking behaviors, Leiser and Moxon have found that all investigated neurons fire during whisking in air with a broad spectrum of frequencies centered around *f*_w_. They also evidenced correlations between the whisking frequency *f*_w_ and the firing rate. The elicited responses due to the whisking in air could define a noise baseline as in our biomimetic experiment. Interestingly, they were also able to differentiate the neural response in the TG arising from SA and FA neurons. They show that SA neurons fire at higher rates (≈ 16 Hz) than FA neurons (≈ 6 Hz) for the whisking in air. Once whiskers contacts occur, the mean firing rate of FA neurons increases by nearly a factor 20, whereas the one of SA neurons only by a factor 6. Last, both in air and in contact, distributions of firing rates of SA neurons largely overlap, whereas those of FA neurons are separated [see Figure 5D of Leiser and Moxon (2007)]. The contact induced response of SA neurons that are sensitive to the overall mechanical stress acting on the base, is therefore rather “polluted” by the whisking phase in air. Since responses of SA neurons increase with the intensity of the stimulus, one may thus expect that a contact will be better detected by SA neurons when mechanical stresses are stronger, so that contrast with the whisking in air becomes larger. This likely occurs typically when the contact point is close to the base and the event occurs at the beginning of the protraction (i.e., ϵ → 1, φ_*C*_ → −φ_0_).

The behavior of FA neurons is fundamentally different. FA mechanoreceptors are sensitive to rapidly time varying stress signals. For humans, a class of FA mechanoreceptors (Pacinian corpuscles) operate a bandpass filtering of the mechanical input around 250 Hz. Interestingly, this frequency is close to the resonance frequency of the skin (Manfredi et al., 2012). By analogy, FA mechanoreceptors of rodents likely have an optimal response around the whisker's resonance frequency (Andermann et al., 2004). Recent Voltage Sensitive Optical Imaging (VSDi) experiments have also shown that the neural activity in the barrel cortex, in response to oscillatory excitation of the whisker was enhanced when the excitation frequency is about 300 Hz (Tsytsarev et al., 2016). Since *f*_w_ is much lower than *f*_0_ by more than an order of magnitude, one may expect that whisking in air will trigger less activity in FA neurons. It has indeed been shown that FA neurons display more quiescent periods than SA neurons during whisking in air (Leiser and Moxon, 2007). However, the precise shape of the filter operated by FA mechanoreceptors in rodents is to our knowledge not precisely evidenced. We have therefore used a first order bandpass filter centered around the vibration frequencies of whiskers. The robustness of the dynamical detection mode was found to remain stable when tuning the filter width and systematically allowed a better detection of first contacts, in timescales of about a few milliseconds. This robust timing of contact detection carried by the dynamical mechanical signal might be the relevant input for spike timing strategies.

Finally, let us note that there is an additional advantage in having $\tau_{\tilde{M}}$ constant, as provided by the vibration based method, to measure the *angular* position of the object. Electrophysiological measurements (Leiser and Moxon, 2007) suggest that the angle of the whisker during a whisking cycle is encoded at the neuronal level, ensuring angular proprioception. Interestingly, since $\tau_{\tilde{M}}$ weakly depends on the exploratory conditions and is much smaller than 1/*f*_w_, the instant of contact can be accurately determined within the whisking cycle, allowing angular determination. With the total torque signal, τ_*M*_ is not constant and such angular determination is likely less accurate. This angular determination is in fact required for any radial distance determination.

In human digital tactile perception, Johansson and Flanagan (2009) have emphasized the importance of detecting transitions between different types of exploration phases (first contacts, detachment, slippage…). In the context of rodents tactile perception, our work shows that the use by the animal of the vibrational mechanical stress at the base of the whisker provides a more efficient, accurate and robust detection of such transitions. Indeed this vibrational component defines a quasi-constant time delay between contact and detection, weakly dependent on the exploratory conditions. Moreover, due to the marked decoupling between whisking and vibration frequencies, the vibrational based detection is less sensitive to mechanical perturbations arising from the whisking motion. With such a scenario, first contacts, for which no prior knowledge of the contact parameters are known by the animal, are likely to be detected by FA mechanoreceptors. However, a parallel detection, involving also SA mechanoreceptors and thus a continuous measurement of stresses is highly probable. Indeed, such a parallel processing, eased by a frequency decoupling, would further increase the detection capabilities, as in human digital tactile perception (Scheibert et al., 2009). Taken all together, our results call for experiments on real rodents, combining slow/fast mechanical base torque measurements associated with electrophysiological measurements.

## Author contributions

GD, AP, and EW designed the research program. LC did the experiments. LC, YB, and GD did the theoretical work. AP and EW wrote the paper.

## Funding

Financial support from both C'Nano Île de France (Project Biostar) and Emergence Ville de Paris (Project SyTaRes) is gratefully acknowledged.

### Conflict of interest statement

The authors declare that the research was conducted in the absence of any commercial or financial relationships that could be construed as a potential conflict of interest.
