# Supplementary material for: Whisker Contact Detection of Rodents Based on Slow and Fast Mechanical Inputs
Source: Front Behav Neurosci. 2017 Jan 10;10:251. doi: 10.3389/fnbeh.2016.00251 (PMC5222834; doi:10.3389/fnbeh.2016.00251)
Supplement: Supplementary file 1 [file Presentation1.PDF]

Supplementary Material: Whisker contact detection of rodents based on slow and fast mechanical inputs

L. N. Claverie [1], Y. Boubenec [2], G. Debrégeas [1], A. Prevost [1] and E. Wandersman [1,*]

*Correspondence:
E. Wandersman, Laboratoire Jean Perrin, Université Pierre et Marie Curie, 4, Place Jussieu F-75005, Paris, France
elie.wandersman@upmc.fr

1 COMPUTING CONICAL EIGENMODES USING A CYLINDRICAL DECOMPOSITION

The spatial eigenmodes V_i of an elastic cone (and associated eigenfrequencies ω_i) are obtained using the Ritz method (Timoshenko, 1937), following an unpublished work by Svodoba and coworkers. The principle of this method consists in decomposing a given conical eigenmode V_i into a sum of n cylindrical eigenmodes Ψ_p

$$V_i(s) = \sum_{p=1}^{n} \alpha_p \Psi_p(s) \tag{S1}$$

The method thus requires the computation of the cylindrical eigenmodes Ψ_p and their corresponding eigenfrequencies λ_p. These are obtained by solving Euler-Bernoulli's equation for a cylinder given by

$$y^{(4)}(s,t) + k_0^2\, \ddot{y}(s,t) = 0 \tag{S2}$$

where the double dot symbol $\ddot{}$ stands for the double time derivative $\partial^2/\partial t^2$ and the exponent (4) for the fourth spatial derivative $\partial^4/\partial s^4$. The characteristic timescale k_0 is defined as $k_0^2 = 4\rho L^4/(Eb^2)$. We solve Equation S2 by seeking a solution in the form of a standing wave $y(s,t) = \sum_j \Psi_j(s) r_j(t)$. This yields the following set of equations

$$\begin{aligned} \Psi_j^{(4)}(s) - \beta_j^4\, \Psi_j(s) = 0 \\ \ddot{r}_j + \lambda_i^2 r_j = 0 \end{aligned} \tag{S3}$$

where $\beta_j^4 = k_0^2\, \lambda_j^2$. The set of Eqns S3 is solved using the same boundary conditions as the ones used for the elastic cone, namely $\Psi_j(1) = 0$, $\Psi_j^{(1)}(1) = 0$, $\Psi_j(\epsilon) = 0$ and $\Psi_j^{(2)}(\epsilon) = 0$, where the exponents (1) and (2) denote respectively $\partial/\partial s$ and $\partial^2/\partial s^2$. The possible values of β_j are solutions of

$$\tan(\beta_j \eta) = \tanh(\beta_j \eta) \tag{S4}$$

where $\eta = 1 - \epsilon$. This yields an analytical solution for the eigenmodes (Timoshenko, 1937) as follows

$$\Psi_j(s) = A\left[\sin\left(\beta_j(1-s)\right) - \mathrm{sh}\left(\beta_j(1-s)\right) - \tan\left(\beta_j(1-\varepsilon)\right)\cos\left(\beta_j(1-s)\right) - \mathrm{ch}\left(\beta_j(1-s)\right)\right)\right] \quad \text{(S5)}$$

where the value of the prefactor A is determined using the normalization condition $\int_\varepsilon^1 \Psi_j^2(s)ds = 0$.
To obtain the α_p coefficients of Eq. S1, the Ritz method is applied (Timoshenko, 1937). It consists in minimizing the total elastic energy associated with the vibration of the cone. The energy $\mathcal{E}(i)$ of the i th eigenmode is defined as

$$\mathcal{E}(i) = \int_\varepsilon^1 [I(s)E(V_i''(s))^2 - \omega_i^2 L^4 \mu(s) V_i(s)^2]ds \quad \text{(S6)}$$

with $I(s) = (\pi b^4 s^4)/4$ the area moment of inertia, and $\mu(s) = \rho\pi b^2 s^2$ the mass per unit length. Defining $\omega_i'^2 = 4\omega_i^2 L^4 \rho/(Eb^2) = k_0^2\omega_i^2$, Equation S6 now writes

$$\mathcal{E}(i) = \int_\varepsilon^1 [s^4(V_i^{(2)}(s))^2 - \omega_i'^2 s^2 V_i^2(s)]ds \quad \text{(S7)}$$

The energy $\mathcal{E}(i)$ is then minimized with respect to α_p

$$\frac{\partial}{\partial\alpha_p}\left[\int_\varepsilon^1 s^4(\sum_p \alpha_p \Psi_p^{(2)}(s))^2 - \omega_i'^2 s^2(\sum_p \alpha_p \Psi_p(s))^2]ds\right] = 0 \quad \text{(S8)}$$

which gives

$$\sum_{m=1}^{n} \alpha_m[\int_\varepsilon^1 [s^4 \Psi_m^{(2)}(s)\Psi_p^{(2)}(s) - \omega_i'^2 s^2 \Psi_m(s)\Psi_p(s)]ds] = 0 \quad \text{(S9)}$$

This equation must be verified for each cylindrical eigenmode Ψ_p ($p \in [1, n]$). One can therefore define a matrix $A_{p,m}$ as

$$A_{p,m}(\omega_i') = \int_\varepsilon^1 [s^4 \Psi_m^{(2)}(s)\Psi_p^{(2)}(s) - \omega_i'^2 s^2 \Psi_m(s)\Psi_p(s)]ds \quad \text{(S10)}$$

with the condition $\det(A)$=0. The roots of Eqn S10 give the possible ω_i' and thus the ω_i. Once the ω_i are known, Eqn S9 is written for every Ψ_p, with $p \in [1, n]$, leading to a system of n equations with n unknown variables, which are the α_m. The coefficients α_m are then determined and give the spatial eigenmode V_i. Note that this mode has to be normalized as follows

$$\int_\varepsilon^1 s^2 V_i^2(s)ds = 1 \quad \text{(S11)}$$

To compute conical eigenmodes, we used $n = 11$ cylindrical eigenmodes. We were able to compute the five first conical eigenmodes for a contact point ϵ varying from 0.02 to 0.7. To test the validity of the cylindrical decomposition method, we computed and compared the conical eigenmodes using on one hand the direct resolution of Euler-Bernoulli's equation for a cone as performed in (Boubenec et al., 2012) and

on the other hand the same eigenmodes obtained using the cylindrical decomposition method. We present on Fig. S1 such comparison for $\epsilon = 0.1$, for the first four eigenmodes. The spatial eigenmodes obtained with both methods overlap very well, which validates the cylindrical decomposition method described here.

2 MOVIE OF A BIOMIMETIC WHISKING EXPERIMENT

A movie of a biomimetic whisking experiment is provided as a supplementary material (textsee“Movie_whisking_Supplemental.m4v”) . On the left panel of the movie, a natural C1 rat whisker oscillates around the rheometer axis at a frequency fw=15 Hz and amplitude ?0=10, to mimic the whisking motion of rodents. To reproduce the approach of rodents towards an obstacle, a sharp object (a wedge) is mounted on a linear motorized translation stage and moved at constant velocity towards the whisker. Optical imaging is performed with a fast camera operating at 25000 frames per seconds. The white bar is 1 cm long. Image analysis is performed (see the text) to extract the base curvature of the whisker and to deduce the base torque. The latter is plotted as a function of time in the right panel of the movie.

The initial position of the wedge is out of reach of the whisker, so that the first phase of the experiment consists in a free whisking in air. The indenter is then translated at a constant velocity towards its final position where it is stopped. The last phase of the experiment thus consists of multiple successive contacts with the fixed indenter.

REFERENCES

Timoshenko S. Vibration Problems in Engineering, New York (1937).

Boubenec Y, Shulz DE, Debrégeas G. Whisker encoding of mechanical events during active tactile exploration. *Frontiers in Behavorial Neuroscience* **6** (2012) 1–12.

3 FIGURES

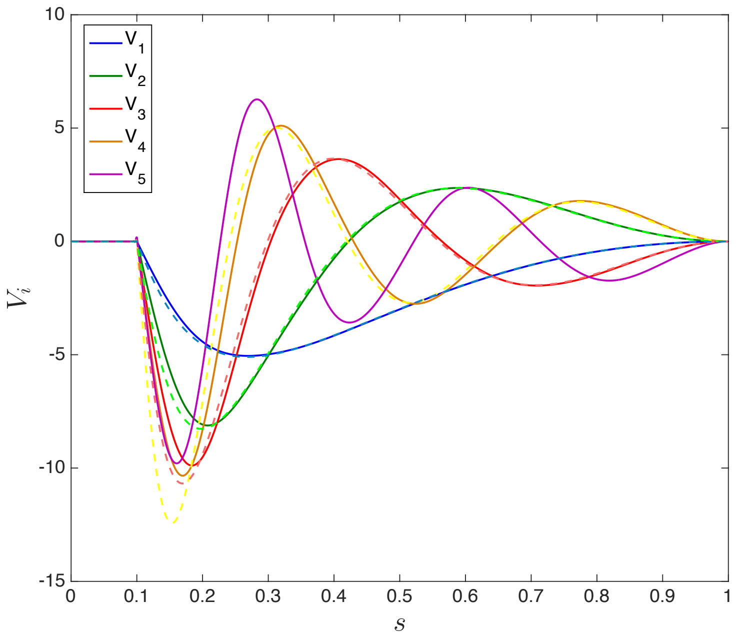

Figure S1. Spatial eigenmodes of an elastic cone in contact at $\epsilon = 0.1$. Blue, green, red, orange and purple colors correspond respectively to the first, second, third, fourth and fifth eigenmodes. The dotted lines correspond to the spatial eigenmodes computed using the direct numerical resolution of Euler-Bernoulli's equation for a cone. This method can be used up to the fourth eigenmode. The solid lines correspond to the spatial eigenmodes computed using the cylindrical decomposition over $n = 11$ cylindrical modes.
